# Near-Complete Genome Sequences of Three Foot-and-Mouth Disease Virus O/ME-SA/Ind-2001e Isolates Obtained from Cattle and Pigs in Thailand in 2016

**DOI:** 10.1128/mra.01110-22

**Published:** 2023-01-18

**Authors:** Katsuhiko Fukai, Tatsuya Nishi, Tomoko Kato, Rie Kawaguchi, Kingkarn Boonsuya Seeyo, Kazuki Morioka

**Affiliations:** a Kodaira Research Station, National Institute of Animal Health, National Agriculture and Food Research Organization, Kodaira, Tokyo, Japan; b Regional Reference Laboratory for Foot and Mouth Disease in South East Asia, Pakchong, Nakhon Ratchasima, Thailand; DOE Joint Genome Institute

## Abstract

Here, we report near-complete genome sequences of three foot-and-mouth disease viruses isolated in 2016 from bovine and porcine epithelial tissue samples collected in Nonthaburi, Songkhla, and Ratchaburi provinces, Thailand. These viruses were classified as serotype O, topotype ME-SA, and sublineage Ind-2001e.

## ANNOUNCEMENT

Foot-and-mouth disease (FMD) virus (FMDV; *Aphthovirus*, *Picornaviridae*) causes FMD, which is the most contagious disease of cloven-hoofed mammals, characterized by vesicles on the feet and in oral cavities (1). FMD is endemic in Asia, the Middle East, and Africa (2). The single-stranded FMDV genome is a positive-sense 8,400-base RNA. Seven serotypes (O, A, C, Asia 1, and SAT 1 to 3) have genetically divergent topotypes, lineages, and sublineages (3). Although serotype O in Southeast Asia comprises the Cathay, Southeast Asia, and Middle East-South Asia (ME-SA) topotypes (2), the Ind-2001e sublineage within the ME-SA topotype is currently the most predominant (4).

FMDVs O/TAI/269-2/2016, O/TAI/315/2016, and O/TAI/317-3/2016 were isolated in 2016 from animals with typical FMD symptoms in Thailand by using primary lamb kidney cells and then passaged three times in ZZ-R 127 and IB-RS-2 cells (5, 6) (Table 1). Once samples were collected, viruses were isolated immediately and stored at −80°C until RNA extraction. RNAs were extracted from cell culture supernatants using a High Pure viral RNA kit (Roche Diagnostics). Near-complete viral genomes (approximately 7.7 kb) were amplified using a SuperScript IV one-step reverse transcription-PCR (RT-PCR) system (Life Technologies) and two FMDV-specific primer sets (7). Libraries were prepared using an Ion Xpress Plus fragment library kit (Life Technologies). DNAs were fragmented for 8 min to generate average 300-bp fragments, which were barcoded using Ion Xpress barcodes (Life Technologies). Libraries were sequenced using an Ion Torrent PGM sequencer (Life Technologies), 314v2 chip, and Ion PGM sequencing 200 kit version 2 (Life Technologies). Torrent suite software version 5.0.5 (Life Technologies) yielded total reads as follows: 67,779 (15,198,038 bp), O/TAI/269-2/2016; 44,701 (9,639,561 bp), O/TAI/315/2016; and 32,699 (6,594,969 bp), O/TAI/317-3/2016. These were assembled and mapped using the PathogenAnalyzer software version 1.2 with default parameters (minimum coverage, 15; minimum coverage for mixed calls, 40; minimum coverage for indels, 40; minimum fraction for mixed calls, 0.2; minimum fraction for out-of-frame indels, 0.75) (Life Technologies). Final assemblies of O/TAI/269-2/2016, O/TAI/315/2016, and O/TAI/317-3/2016 comprised 7,692, 7,692, and 7,694 nucleotides (nt); 53.45%, 53.52%, and 53.46% G+C contents; and 1,976, 1,253, and 857.4 average coverage depths, respectively.

**TABLE 1:** Information about isolates

Isolate	Province	District	Subdistrict	Species	Lesion site	Collection date (day/mo/yr)
O/TAI/269-2/2016	Nonthaburi	Bang Khu Rat	Bang Bua Thong	Cattle	Lip	13/10/2016
O/TAI/315/2016	Songkhla	Sadao	Padang Besor	Cattle	Tongue	8/11/2016
O/TAI/317-3/2016	Ratchaburi	Ban Pong	Nong Or	Pig	Nose	10/11/2016

**FIG 1:**
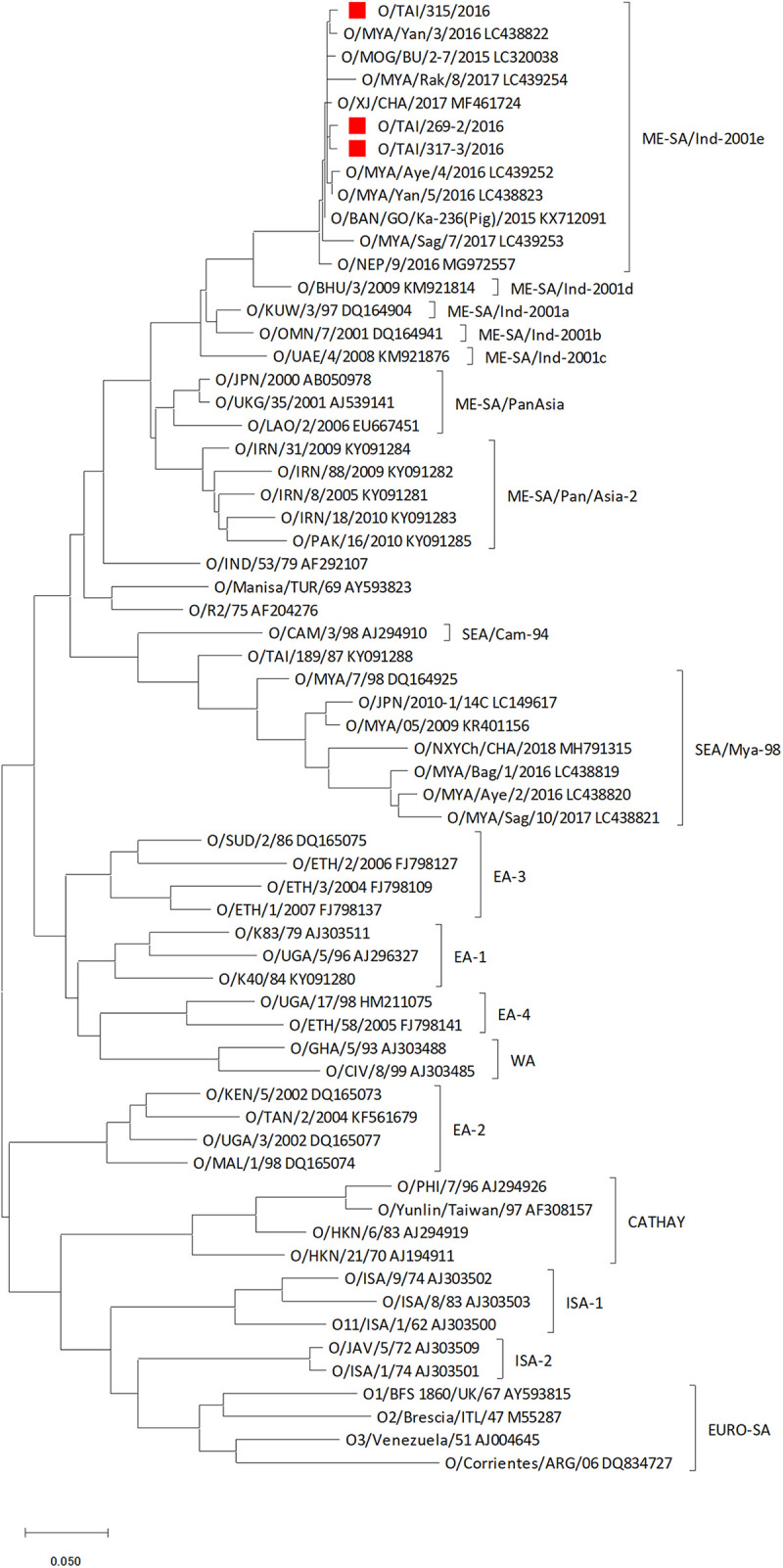
Phylogenetic analysis of VP1 sequences of three FMDV isolates in this study. VP1 sequences of FMDV isolates were used for phylogenetic analysis using a maximum likelihood method with default parameters in MEGA version 10.2.0 (https://www.megasoftware.net/). Horizontal distances are proportional to the minimum number of nucleotide differences required to join nodes and sequences. The isolates in this study are marked by red squares. The reference strains of each topotype/lineage are shown along with GenBank accession numbers after each strain name.

Genome sequences of O/TAI/269-2/2016, O/TAI/315/2016, and O/TAI/317-3/2016 were the most closely related to those of O/VIT/20/2016 (GenBank accession number MG983741) (8), O/MYA/Yan/3/2016 (GenBank accession number LC438822) (9), and O/VIT/20/2016 (8) with 99.64%, 99.25%, and 99.47% nucleotide sequence identity rates, respectively, according to NCBI BLASTn analysis (analyzed on 18 October 2022 at http://blast.ncbi.nlm.nih.gov/Blast.cgi). These genomes were classified as O/ME-SA/Ind-2001e by phylogenetic analysis together with sequences obtained from the GenBank database (Fig. 1).

The near-complete genome sequences of three O/ME-SA/Ind-2001e FMDVs determined here will contribute to efforts to monitor epidemiological information concerning outbreaks and to implement appropriate countermeasures.

### Data availability.

The nucleotide sequences of the three FMDVs reported here were deposited in GenBank under accession numbers LC729870, LC729871, and LC729872. Raw sequence reads were deposited in the DDBJ Sequence Read Archive (DRA) under BioProject accession number PRJDB14377 and DRA Run accession number DRR408791 (O/TAI/269-2/2016), BioProject accession number PRJDB14378 and DRA Run accession number DRR408792 (O/TAI/315/2016), and BioProject accession number PRJDB14379 and DRA Run accession number DRR408793 (O/TAI/317-3/2016).
